# Metabolite Changes Associated with Resectable Pancreatic Ductal Adenocarcinoma

**DOI:** 10.3390/cancers17071150

**Published:** 2025-03-29

**Authors:** Declan McDonnell, Paul R. Afolabi, Umar Niazi, Sam Wilding, Gareth O. Griffiths, Jonathan R. Swann, Christopher D. Byrne, Zaed Z. Hamady

**Affiliations:** 1 Human Development & Health, University of Southampton, Southampton SO16 6YD, UK; p.r.afolabi@soton.ac.uk (P.R.A.); u.niazi@soton.ac.uk (U.N.); z.hamady@soton.ac.uk (Z.Z.H.); 2 Department of General Surgery, University Hospital Southampton NHS Foundation Trust, Southampton SO16 6YD, UK; 3 Cancer Research UK Southampton Clinical Trials Unit, University of Southampton, Southampton SO16 6YD, UK

**Keywords:** pancreatic, adenocarcinoma, PDAC, metabolite, metabolomics

## Abstract

Introduction: Pancreatic ductal adenocarcinoma (PDAC) is insidious, with only 15–20% of those diagnosed suitable for surgical resection as it is either too advanced and has invaded local structures or has already spread to distant sites. The associated tumor microenvironment provides a protective shield which limits the efficacy of chemotherapeutic agents, but also impairs the delivery of nutrients required for the PDAC cells. To compensate for this, metabolic adaptions occur to provide alternative sources of fuel. The aim of this study is to explore metabolomic differences between participants with resectable PDAC compared to healthy volunteers (HV). The objectives were to use nuclear magnetic resonance (NMR) spectroscopy and mass spectrometry (MS) to determine if resectable PDAC induces sufficient metabolic adaptations and variations which could be used to discriminate between the two groups. Methods: Plasma samples were collected from fasted individuals with resectable PDAC (*n* = 23, median age 68 [IQR 56–75], 69.6% male) and HV (*n* = 24, median age 63 [IQR 58–71], 54.2% male). Samples were analyzed using NMR and the Biocrates MxP Quant 500 kit at University Hospital Southampton. Results: NMR spectroscopy identified six independent metabolites that significantly discriminated between the PDAC and HV groups, including elevated plasma concentrations of 3-hydroxybutyrate and citrate, with decreased amounts of glutamine and histidine. MS analysis identified 84 metabolites with a significant difference between the PDAC and HV cohorts. The metabolites with a fold change (FC) > 1.5 in the PDAC population were conjugated bile acids (taurocholic acid, glycocholic acid, and glycochenodexoycholic acid). Discussion: In conclusion, using metabolomics, biochemical differences between resectable PDAC and HV were detected. These differences indicate metabolic plasticity and utilization of alternative fuel sources.

## Simple Summary

Pancreatic ductal adenocarcinoma (PDAC) has a high mortality rate and is rarely suitable for curative treatment due to late presentation. Endocrine and exocrine dysfunction of the pancreas is common, which presents with increased blood glucose levels and restricted production of digestive enzymes. These are direct consequences of PDAC-induced insulin resistance, and mechanical obstruction of the pancreatic ducts, respectively. The microenvironment of this tumor provides a protective cocoon that shields PDAC cells from therapeutic agents, but also limits their nutrient availability. To compensate for these disturbances, metabolic adaptations occur to promote and ensure cell survival through the provision of alternative fuel sources. This study uses different analytical techniques to evaluate a cohort of participants with resectable PDAC compared to healthy volunteers to demonstrate that several of these changes occur early, when the cancer is still suitable for surgical resection.

## 1. Introduction

Pancreatic ductal adenocarcinoma (PDAC) is the third most common cause of cancer death after lung and colorectal cancers [1]. Surgery is only indicated in the earliest stages, but given the aggressive nature of PDAC, only 15–20% of patients at the time of their diagnosis are suitable for surgical resection; most patients will have locally advanced disease or evidence of distant metastases [2]. Overall, PDAC has a dismal 5-year survival rate of 12%; however, in those suitable for surgical resection, the 5-year survival rate doubles to 24% [3]. One of the key features of PDAC that contributes to the challenge of treating it successfully with chemotherapy is the desmoplastic tumor microenvironment (TME). Within the TME, cancer-associated fibroblasts are induced by PDAC cells, under the influence of a *KRAS* oncogene, to deposit excessive amounts of connective tissues, such as collagen, resulting in the bulk of the tumor volume being composed of stroma [4]. This results in the PDAC cells having a dense, protective cocoon resistant to penetration by chemotherapeutic agents [5]. A consequence of this desmoplastic stroma protecting the PDAC cells is that the TME is hypoxic and devoid of nutrients [5], a status aggravated by the impaired cellular glucose availability due to systemic insulin resistance associated with PDAC [6]. This necessitates a degree of metabolic plasticity, influenced by *KRAS*, to promote alternative pathways in order to meet these demands and to ensure an optimal cellular redox state [7,8].

A variety of chemotherapeutic agents can be used in the treatment of PDAC to impair cell growth, with resistance to these agents occurring via two pathways: intrinsic and acquired [9]. Intrinsic resistance signifies treatment is ineffective from the beginning. Acquired resistance is likely to arise given the progressively dense TME. This physical barrier also impairs other treatment modalities, such as immunotherapy. Novel treatments, such as focal adhesion kinase inhibitors and immune checkpoint inhibitors, can act as adjuncts and impair the formation of the fibrotic and immunosuppressive PDAC TME as they mediate interactions between collagen and integrins to activate downstream signaling cascades [10,11,12,13,14]. Similar alternative strategies include utilizing pirfenidone to impair pancreatic stellate cells, coupled with a *KRAS*-specific inhibitor (AMG510) and the traditional chemotherapy agent gemcitabine [15].

Metabolomics is the study of processes which result in detecting altered concentrations of byproducts of metabolism. The principal analytical techniques used in studying metabolomics are nuclear magnetic resonance (NMR) spectroscopy and mass spectrometry (MS), with the latter used in around 70% of published metabolomic studies [16]. These can be used to highlight biochemical derangements associated with certain diseases, such as the variations in amino acids and lipids associated with PDAC [17]. The most common deviation in serum and plasma metabolite concentrations associated with PDAC arise from glutaminolysis: an increased amount of glutamate and corresponding decreased amount of glutamine [18]. If required, glutamate can be subsequently metabolized into α-ketoglutarate to maintain the tricarboxylic acid (TCA) cycle. This process reliably takes place in resectable PDAC [19,20,21,22]. It is currently unclear which other altered metabolite concentrations could be used to demonstrate the presence of an underlying resectable PDAC compared to healthy volunteers (HV). One example is the ketone body 3-hydroxybutyrate (3-HB), which has been demonstrated to have increased [22] and decreased [19,23] levels detected in the blood tests of patients with PDAC compared to non-PDAC controls.

The aim of this novel study is to perform a comprehensive untargeted metabolomic analysis of plasma from fasted participants with resectable PDAC and HV. The objectives are to use NMR and liquid chromatography–mass spectrometry (LCMS) to characterize the metabolic phenotypes of each cohort. This analysis was designed to provide the basis for developing a distinct metabolic biochemical signature for resectable PDAC, which could eventually be developed into a biomarker test to facilitate early detection of, and improve overall survival in, PDAC patients.

## 2. Materials and Methods

### 2.1. Study Design

This case-control pilot study was conducted at the Southampton Clinical Trials Unit (SCTU) within the University Hospital Southampton NHS Foundation Trust (UHSFT). It was conducted against patients with resectable PDAC and compared to HV. The Health Research Authority and Health and Care Research Wales provided the ethical approval (REC reference 20/NS/0105, IRAS project ID: 286297, on 21 October 2020). Statistical support was provided by SCTU. This was a Cancer Research UK-funded project, and the protocol is already published [24].

### 2.2. Participants

Participants were recruited from the regional catchment area covering the UK counties of Hampshire and the Isle of Wight, as well as Dorset, and the Channel Islands. Both male and female participants were invited, between the ages of 30 and 85. Those participants with resectable PDAC were determined to be suitable for recruitment to the study following discussion at the regional multi-disciplinary team (MDT) meeting at UHSFT. Every patient with PDAC underwent a computer tomography (CT) scan of their chest, abdomen and pelvis as the routine standard investigation. Positron emission tomography (PET) or liver magnetic resonance imaging (MRI) were undertaken if there was any diagnostic doubt about possible metastatic disease and the patient’s suitability for resection. Patients were excluded from this study if there was any evidence of metastatic disease detected on CT, PET, or MRI. Eligible PDAC participants were recruited following the MDT decision that they had resectable disease. They were recruited following consultation with a surgeon to inform them of their diagnosis. They were recruited to the study prior to commencing any chemotherapy regimens. HV participants were recruited from the Project Management Recruitment Team based at UHSFT, as well as willing friends and family members of the PDAC participants who were also keen to participate in this study. Each individual was given an appropriate participant information sheet explaining the research and signed an informed consent form.

### 2.3. Sample Collection and Preparation

The study was conducted in the National Institute for Health and Care Research (NIHR) Southampton Clinical Research Facility (CRF) at UHSFT. Participants were required to fast overnight for at least 12 h prior to undergoing a separate ^13^C-mixed triglyceride breath test study [25]. Blood was collected from each fasted participant in a 10 mL lithium heparin BD vacutainer prior to consuming the test meal. These were immediately centrifuged at 600× *g* for 10 min. The clear supernatant plasma was transferred to 15 mL polypropylene centrifuge tubes and centrifuged at 1000× *g* for 10 min at room temperature. This supernatant was transferred to a clear 15 mL polypropylene tube before 1 mL aliquots were transferred into 2 mL cryotubes using a pipette. They were then stored upright at −80 °C.

Plasma samples were prepared for NMR analysis based on the method described by Nagana Gowda et al. [26] after all the participants had been recruited. Samples were thawed at room temperature, and 300 µL was combined with 600 µL of methanol chilled to −80 °C and vortexed. Following incubation at −20 °C for 30 min, samples were centrifuged at room temperature for 30 min at 3000× *g* to isolate low-molecular metabolites. The supernatant (600 µL) was transferred to an Eppendorf tube and dried overnight in a vacuum concentrator held at 30 °C. Prior to NMR analysis, the dried samples were reconstituted with 600 µL of phosphate buffer (100% D_2_O solution; NaH_2_PO_4_, Na_2_HPO_4_, NaN_3_ (sodium azide) containing 1 mM of the internal standard, 2,2,3,3-D4-3-(trimethylsilyl)propionic (TSP) acid. Following mixing by vortex for 10 s, 550 µL of this solution was transferred to a 5 mm NMR tube for analysis. One-dimensional proton NMR spectra were acquired for all samples using a Bruker Avance III 700 MHz FT-NMR Spectrometer with a cryoprobe (Bruker Corporation, Billerica, MA, USA). Standard one-dimensional NMR spectra with water suppression were acquired for each sample (32 scans, 4 dummy scans, acquisition time, mixing time, 64 K data points, 20 ppm spectral width).

Separate plasma samples underwent analysis using the Biocrates MxP Quant 500 kit (Biocrates Life Science AG, Innsbruck, Austria) following the manufacturer’s protocol. Lipids and hexoses were measured by flow injection analysis–tandem mass spectrometry (FIA-MS/MS), and small molecules were measured by liquid chromatography–tandem mass spectrometry (LC-MS/MS). Here, 10 µL of plasma samples were pipetted onto the wells containing the inserts and dried under a nitrogen stream using a positive-pressure manifold (Waters). Next, 50 µL of a 5% phenylisothiocyanate solution was added to each well to derivatize amino acids and biogenic amines. After 1 h incubation time at room temperature, the plate was dried again. To extract the metabolites, 300 µL 5 mM ammonium acetate in methanol was pipetted to each filter and shaken for 30 min at room temperature. The extract was eluted into a new 96-well plate using positive pressure. The pipetting order of samples was randomized before application onto the 96-well plates. For further LC-MS/MS analyses, 150 µL of the extract was diluted with an equal volume of ultra-pure water. For FIA-MS/MS analyses, 10 µL extract was diluted with 490 µL of flow injection analysis (FIA) solvent (provided by Biocrates). After dilution, the obtained extracts were then analyzed by LC-MS/MS and FIA-MS/MS methods using multiple reaction monitoring (MRM) to detect the analytes. This system comprised of an Accuity Premier ultra-performance liquid chromatography (UPLC) with Sample Manger FTN (Waters) system coupled with a Waters XEVO-TQXS mass spectrometry system (Waters Corp., Wilmslow, UK) in electrospray ionization (ESI) mode. Metabolites were analyzed via LC-MS in positive and negative mode. In addition, 5 µL of the sample extract were injected to the ultra-high performance liquid chromatography (UHPLC) column at 50 °C using a 5.8 min solvent gradient employing 0.2% formic acid in water and 0.2% formic acid in acetonitrile. Sample extracts (20 µL) were used in the FIA of metabolites in the positive and negative mode. All FIA injections were carried out using the Biocrates FIA solvent. All metabolites were identified and quantified using isotopically labelled internal standards and MRM.

### 2.4. Statistical Methods

Normality was established using the Skewness–Kurtosis function within Stata 16. If data had a normal distribution, it was reported as means (standard deviation). Medians (interquartile range) were used for data that did not have a normal distribution.

For NMR analysis, the Fourier-transformed spectra were automatically corrected for phase and baseline distortions and calibrated to TSP in Topspin 4.07 (Bruker). Spectra were imported into Matlab (version 2018a, MathWorks) using in-house scripts, and resonances arising from water, urea. and methanol were removed. Principal component analysis (PCA) models were constructed using in-house scripts and the scores plots were used to check for outliers. Full-resolution NMR spectra were interrogated using orthogonal projection to latent structures–discriminant analysis (OPLS-DA). Here, pairwise models were built to compare the biochemical profiles of the different groups (PDAC, HV). A seven-fold cross-validation approach was used to assess the predictive performance (Q^2^Y) of the OPLS-DA model (i.e., how well the NMR data predicts class outcome) [27]. The empirical significance of the Q^2^Y parameter was evaluated using a permutation testing approach with 1000 iterations. For a model to be significant, the Q^2^Y value for the true model must be in the top 5% of values generated from the permuted models. The degree of significance was set at a 95% value (0.05). Spectral peaks from valid models were investigated to identify discriminatory features between the groups. The data were visualized by backscaling the covariance of each peak/datapoint with the response variable to display the direction of association. Peaks significantly associated with the response variable (i.e., sample class) were highlighted in red and non-significant regions were plotted in black. To calculate the relative abundance for each discriminatory metabolite, the lower and upper bounds (ppm) of their corresponding spectral peak was identified and the area under the peak between these points was obtained. Regression analysis using the relative abundance of each metabolite was then undertaken and any metabolites that demonstrated multicollinearity (variance inflation factor [VIF] > 4) were removed from the model. This was done to optimize the creation of a biochemical signature panel and ensure the inclusion of independent biomarkers for resectable PDAC. The peak arising from the internal standard and the corresponding protons were then used to calculate approximate concentrations based on the number of protons present in TSP (9). A Mann–Whitney U test was used to compare the difference in the concentrations between the groups. False discovery rate (FDR) was adjusted for using the Benjamini–Hochberg method. Logistic regression analysis was then performed based on these concentrations to compare PDAC vs. HV, with the post-estimation command *predict* used to generate a single combined variable to create an ROC curve to test discrimination between PDAC and HV.

The raw data from the LC-MS and FIA-MS analyses were exported and processed using the Biocrates MetIDQ™ software (version Oxygen-DB110-3023) in accordance with the manufacturer’s protocol. To guarantee the high quality of metabolome data, quality control samples-based data normalization was carried out to minimize the variation of analyses. Initial data cleaning was carried out by excluding metabolites with >90% missing values or values below the limit of detection (LOD) in patients with PDAC and healthy control groups. In addition, all the metabolites with >90% of the concentration values above the LOD in patients with PDAC and healthy control groups were included for statistical analysis. The processed raw data from MetIDQ were converted into a CSV file and subjected to further manual sorting in Microsoft Excel (Microsoft Office 365, Albuquerque, NM, USA). The differential abundance analysis of the LC-MS data is described in Section 3.2. FDR was adjusted for using the Benjamini–Hochberg method.

Spectral analytics was undertaken with Matlab (R2018a, The MathWorks, Natick, MA, USA) [28]. Statistical analyses for NMR data were carried out using Stata 16 (StataCorp, College Station, TX, USA) [29]. Statistical analyses for MS data were carried out using R (https://www.R-project.org (accessed on 14 August 2024)) [30] and Stan (https://mc-stan.org (accessed on 30 August 2024)) [31]. The STROBE cohort reporting guidelines were used throughout.

## 3. Results

### 3.1. Participant Demographics

By the end of the 12-month recruitment period (16 March 2021–16 March 2022). Only those participants who had PDAC confirmed by histology, either following surgical or endoscopic intervention, were included for analysis. This excluded anyone with a neuroendocrine tumor (*n* = 1) or cholangiocarcinoma (*n* = 3). At the end of the study, 23 participants with resectable PDAC were recruited. Within this cohort, 19 had PDAC within the head of pancreas (HOP-PDAC). The median age for the PDAC group was 65 (IQR 56–75) and 15 (68.1%) were male (Table 1). There were 24 participants in the HV group with a median age of 63 (IQR 58–71) and 13 (54.2%) were male. Using the American Joint Committee on Cancer (AJCC) staging system [32], there were nine with stage I disease, seven with stage II disease, and seven with stage III disease. All stage III diseases were established on post-operative histology and were considered resectable at the time of the study. All had a diagnosis of adenocarcinoma except one high grade dysplasia and one acinar cell cancer. These data, along with medication history, are available in Supplementary File S1.

### 3.2. Nuclear Magnetic Resonance Spectroscopy Results

A principal component analysis (PCA) model was constructed on all the spectra acquired from the plasma samples (Supplementary File S2). From the scores plot, one male participant (aged 63 years) with HOP-PDAC was identified as an outlier due to substantially higher amounts of O-acetylcarnitine. This was unique to this individual and it could not be determined if this signal was related to medication, biochemical variability in this individual, or a sample contaminant. As such, this sample was removed from subsequent NMR analyses (Supplementary File S3). This left 22 PDAC participants with a median age 68 (IQR 56–75) years, 68.2% of which were men.

**Figure 1:**
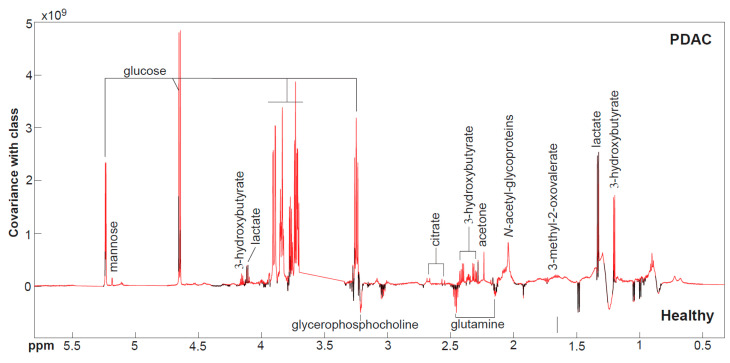
OPLS-DA model comparing the circulating metabolic profiles from participants with pancreatic ductal adenocarcinoma (PDAC) vs. healthy volunteers measured by ^1^H NMR spectroscopy (Q^2^Y = 0.30, *p* = 0.001). The coefficients plot indicates covariance with class, with positive peaks having a greater abundance in PDAC plasma compared to healthy volunteers and negative peaks being present in lower amounts. Red spectral peaks indicate metabolites significantly associated with class membership. PEG, polyethylene-glycol.

The OPLS-DA model constructed between the PDAC and HV groups demonstrated good predictive ability to discriminate between the two groups (Q^2^Y = 0.30, *p* = 0.001) (Figure 1). The metabolites with a greater relative abundance in PDAC compared to HV are: 3-HB, lactate, 3-methyl-2-oxovalerate (3-M-2-O), *N*-acetylglycoproteins (NAG), acetone, citrate, glucose, mannose, 4-hydroxyphenylacetate (4-HPA) and 5 hydroxyindoleacetate (5-HIAA). The metabolites with a lower relative abundance in PDAC are glycerophosphocholine (GPC), glutamine, and histidine. One participant with PDAC was taking metformin for the past three years due to a previous diagnosis of diabetes mellitus. Their plasma lactate level was found to be markedly elevated (3.6 mM) compared to the median (IQR) for the remainder of the PDAC group (2.2 [1.9–2.7] mM). When the effect of metformin was adjusted for the analysis, no significant difference in lactate abundance was observed between the groups, and therefore, this metabolite was subsequently removed from further analysis. After VIF analysis on these metabolites, 3-M-2-O, acetone, GPC, mannose, 4-HPA, and 5-HIAA had evidence of multicollinearity and were excluded from further analyses (Supplementary File S4). The remaining metabolites had an overall VIF of 1.83, and this is available in Supplementary File S5.

The plasma concentrations of each significant metabolite were estimated based on the concentration of TSP and are shown in Table 2. Of the six metabolites identified, glutamine and histidine were reduced in PDAC compared to HV, while the concentrations of the other metabolites are all elevated. The Benjamini–Hochberg method was used to correct for multiple comparisons between HV and PDAC groups and was set at a 1% FDR (Supplementary File S6).

**Figure 2:**
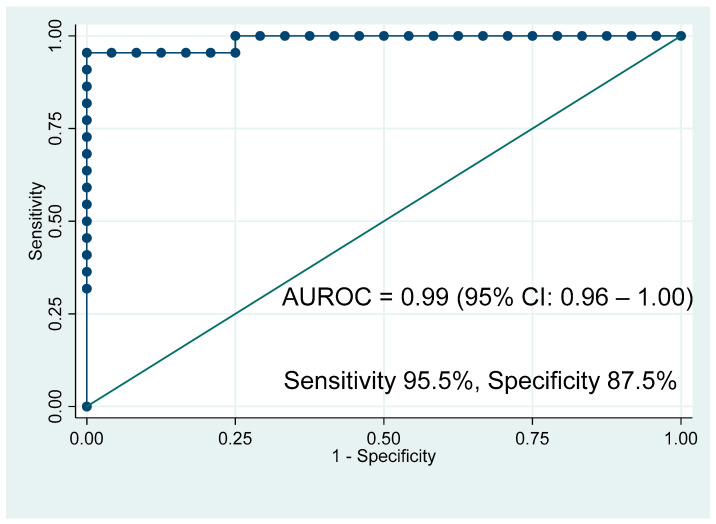
Receiver operating characteristic curve predicting pancreatic ductal adenocarcinoma vs. healthy volunteers based on the metabolomic profile derived from 3-hydroxybutyrate, N-acetylglycoproteins, glutamine, citrate, glucose, and histidine.

To determine if a combined panel of these significant metabolites could discriminate between PDAC and HV, logistic regression was performed using the concentrations of these metabolites. Postestimation analysis was then conducted using the predict var command to produce a one-step-ahead forecast using the estimated coefficient within the endogenous variable [33]. The resultant single variable was used to generate an ROC curve based on this estimation (Figure 2). The AUROC was 0.99 (95% CI: 0.96–1.00), with an estimated sensitivity and specificity of 95.5% and 87.5%, respectively.

### 3.3. Mass Spectrometry Results

In total, 628 metabolites were identified by LC-MS and FIA-MS. Out of the 628 metabolites, 207 metabolites were excluded from further analysis as the number of missing data points was too large to achieve model convergence. A total of 303 metabolites had no missing data, while 118 metabolites had less than 15 missing data in each HV and PDAC group. The missingness normally occurred if the metabolite was below the LOD of the sensor. The exploratory analysis of the metabolite data suggests that the density distribution of the log transformed data had long tails and was modelled using a regression model from the t-distribution family. Furthermore, the variance of the metabolites in the PDAC group was on average higher than HV group, which prompted us to estimate two residual standard deviation terms [34] in the regression model for the 303 metabolites. In the case of the 118 metabolites with missing data, to avoid imputations, a left censored regression model was used as described in the Stan Reference Manual [35]. Model checks were performed using posterior predictive simulations [34]. All *p*-values for log fold difference between HV and PDAC groups were adjusted for multiple testing using the Benjamini–Hochberg method. Using a 1% FDR, 84 metabolites with a significant difference were identified between the HV and PDAC groups (Supplementary File S6). This included a decrease in glutamine and histidine in the PDAC group, consistent with the NMR findings. The heatmap for this analysis is available in Supplementary File S7.

**Figure 3:**
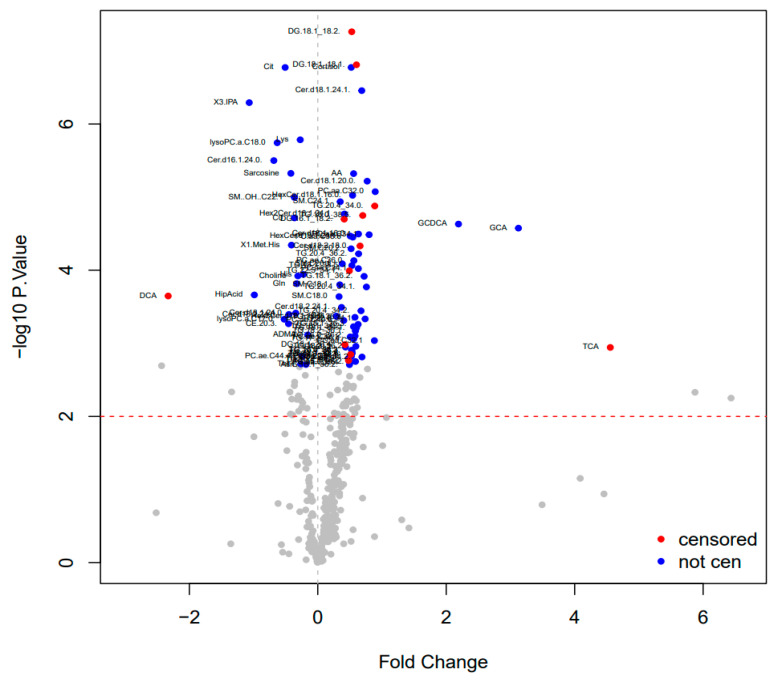
Volcano plot demonstrating the difference in log fold change of metabolites identified using mass spectrometry between participants with pancreatic ductal adenocarcinoma vs. healthy volunteers.

The initial data were processed for metabolites using a natural logarithm for the x axis to measure magnitude of log fold change (FC) (Figure 3). A FC of >±1.5 considered particularly important [36], and those relevant metabolites are displayed in Table 3. Conjugated bile acids have a FC > 1.5 in the plasma of those with PDAC, i.e., taurocholic acid, glycocholic acid, and glycochenodeoxycholic acid. In contrast to this, the unconjugated bile acid deoxycholic acid was significantly elevated in the plasma of HV compared to PDAC.

## 4. Discussion

This study demonstrates extensive differences between the metabolite profiles of participants with resectable PDAC compared to HV. NMR analysis detected changes in the plasma concentrations of six independent metabolites associated with PDAC. The metabolite with the greatest discrepancy between the two groups was glucose, reflecting the fact that hyperglycemia is present in approximately 85% of those with PDAC [37]. Hyperglycemia is a common feature of PDAC due to insulin resistance [38], a paraneoplastic phenomenon induced by PDAC-associated inflammation [39], which often reverses following resection of the PDAC despite the loss of pancreatic parenchyma [40].

To offset the reduced amount of intracellular glucose available, alternative fuel sources are required to maintain cell function. NMR analysis was able to detect evidence of complementary pathways including ketogenesis, in particular the ketone body 3-HB. A compensatory increase in fatty acid oxidation produces an abundance of acetyl-CoA, which is converted to ketone bodies if oxaloacetate levels are depleted, e.g., by gluconeogenesis [41,42]. Each participant was requested to not consume anything other than water for 12 h preceding the test, so this was not a manifestation of fasting. As anticipated, glutaminolysis was also apparent due to decreased amount plasma glutamine as it is converted into glutamate prior to being utilized by PDAC cells [43,44]. This is one of the many *KRAS*-driven hallmarks of PDAC employed to circumvent lack of available nutrients [45]. Another is the use of cytosolic citrate in the formation of phospholipids, following its transportation out of the mitochondria and cleaved by ATP-citrate lysase into acetyl-CoA to produce cholesterol and fatty acids for cell membrane synthesis [46]. This would also increase oxaloacetate usage and result in further ketogenesis [47].

**Figure 4:**
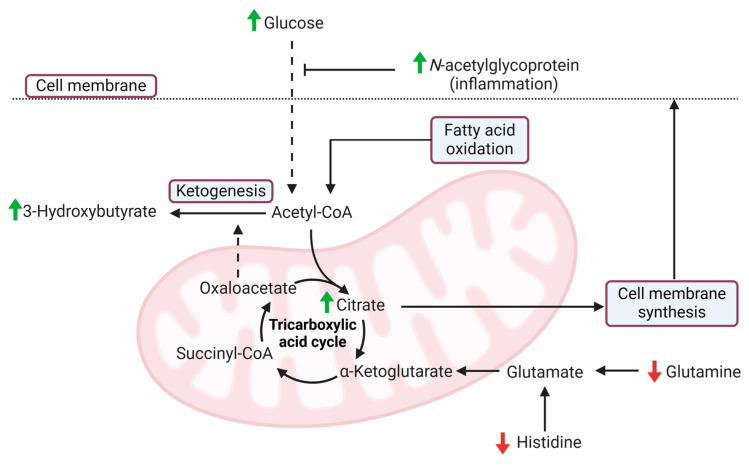
Metabolomic alterations induced by pancreatic ductal adenocarcinoma (PDAC) detected using nuclear magnetic resonance (NMR) spectroscopy. The paraneoplastic effect of PDAC impairs systemic insulin sensitivity, which increases plasma glucose levels. This process is influenced by inflammatory mediators such as the cytokines interleukin-1 and interleukin-6, which also drive the production of *N*-acetylglycoprotein. As a result of reduced intracellular glucose, alternative fuel sources are utilized, such as fatty acid oxidation to produce acetyl-CoA. This is used by the tricarboxylic acid (TCA) cycle, to produce citrate. Mitochondrial citrate can move into the cytoplasm where it is then used in the formation of cholesterol and fatty acids for cell membrane synthesis, a *KRAS*-driven hallmark of PDAC. Glutamine and histidine abundance were both reduced because of their conversion into glutamate, which is used to maintain α-ketoglutarate and propagate the TCA cycle. To maintain alternative fuel sources, ketogenesis occurs in the liver and converts acetyl-CoA into ketone bodies, such as 3-hydroxybutyrate, thus increasing the plasma concentration of this metabolite. This latter process can be upregulated if there is a scarcity of oxaloacetate, as is the case with increased citrate production. Solid arrows represent successful pathways, dashed arrows represent impaired pathways.

Additional findings seen in this NMR study are elevated levels of plasma NAG and decreased histidine. NAG, also known as GlycA, is not a distinct, homogenous molecule, but a composite of several N-acetyl groups belonging to several glycan portions on acute phase proteins, such as α1-acid glycoprotein, haptoglobin, and α1-antitrypsin [48]. It is elevated in the PDAC population in response to inflammatory cytokines, such as IL-1 and IL-6, which reflect the strong association between PDAC development and progression, and inflammatory mediators [49,50,51]. Reduced levels of circulating histidine is one of the most frequently detected metabolomic alterations in PDAC [19,21,22,52,53]. Histamine ammonia lyase is an enzyme involved in the first step of histidine catabolism [54], and has been demonstrated to be overexpressed in PDAC [55]. The main pathway of histidine catabolism is the conversion to glutamate via urocanate [56,57]. The requirement to maintain glutamate to replenish α-ketoglutarate and maintain the TCA cycle explains the decreased plasma abundance of glutamine and histidine in this study. Figure 4 is a schematic which illustrates a proposed mechanism for how these processes are combined.

The major limitation of this NMR study is the lack of a validation cohort, which restricts the classification of this metabolite panel to a biochemical signature rather than a biomarker. Additionally, the AUROC produced by this analysis is influenced by overfitting, where the generated algorithm is trained too specifically to predict the outcomes in a particular study population. These factors limit the generalizability of the NMR study findings, which warrant external validation of this model before further clinical application. The relatively small sample size in each group increases the possibility of a type 2 statistical error arising; however, the results are comparable to other similar studies in this area, including OuYang et al. (*n* = 17) [23], Zhang et al. (*n* = 19) [19], Cao et al. (*n* = 28) [58], and Bae et al. (*n* = 34), who also identified glucose and ketone bodies as key metabolites in PDAC [59].

The data from the LC-MS and FIA-MS portion of the study identified 84 distinct metabolites with a significant difference in concentration between the two groups; however, the only difference with an FC > ±1.5 is the increase in conjugated bile acids in those with PDAC, and the unconjugated bile acid deoxycholic acid was significantly elevated in the plasma of HV compared to PDAC. These changes reflect the impending obstruction as a result of the PDAC occluding the lumen of the biliary system, resulting in a backlog of conjugated bile acids, resulting in jaundice and reduced availability of digestive enzymes entering the gastrointestinal tract. In patients with jaundice, they would undergo dedicated imaging to determine the underlying cause and if there was a sinister cause for this obstruction, limiting the clinical applicability of using these particular metabolites for screening or surveillance purposes.

## 5. Conclusions

There are distinct metabolomic changes associated with resectable PDAC, which indicate the metabolic plasticity used to circumvent insulin resistance and subsequent reduced intra-cellular glucose availability, such as ketogenesis and glutaminolysis. These changes are further augmented by the hypoxic, nutrient-deprived conditions found in the dense TME characteristic of PDAC. The changes detected using NMR can be combined into a single panel that can discriminate between PDAC and HV and could form the basis of a distinct biochemical signature composed of a composite of six metabolite profiles. There are several differences detected using MS, with the most prominent reflecting impending biliary obstruction.

## Figures and Tables

**Table 1:** Initial study participant characteristics.

Participant Characteristics	Healthy Volunteers (*n* = 24)	Pancreatic Ductal Adenocarcinoma (*n* = 22)	*p* Value
Age, Years *,	63 (58–71)	68 (56–75)	0.62
Sex ^†^, Men (%)	13 (54.2%)	16 (69.6%)	0.17
Weight, kg ^#^	81.3 ± 19.9	77.1 ± 9.6	0.70
Body Mass Index, kg/m^2 #^	28.3 ± 6.5	26.0 ± 3.7	0.36

Data are presented as means ± SD ^#^ or medians (IQR) * for normally and non-normally distributed variables, respectively. Variables with dichotomized outcomes are labelled with ^†^. One participant with pancreatic ductal adenocarcinoma was excluded due to high plasma levels of O-acetylcarnitine.

**Table 2:** Plasma concentrations of metabolites measured by nuclear magnetic resonance spectroscopy comparing healthy volunteers to participants with pancreatic ductal adenocarcinoma.

Metabolite	Healthy Volunteers (*n* = 24) (µM)	Pancreatic Ductal Adenocarcinoma (*n* = 22) (µM)	*p* Value
3-Hydroxybutyrate, median (IQR)	374 (309–414)	423 (378–747)	0.019
*N*-Acetylglycoproteins, median (IQR)	462 (426–641)	640 (582–789)	<0.001
Glutamine, median (IQR)	894 (841–954)	809 (723–891)	0.0049
Citrate, median (IQR)	168 (154–193)	213 (178–242)	0.0011
Glucose, median (IQR)	3810 (3585–4215)	4469 (4080–7020)	<0.001
Histidine, median (IQR)	368 (356–396)	323 (270–357)	0.002

**Table 3:** Metabolites with a fold change greater than ±1.5 detected by mass spectrometry in participants with pancreatic ductal adenocarcinoma versus healthy volunteers.

Metabolite	Fold Change	*p* Value
Taurocholic acid	4.55	0.001
Glycocholic acid	3.12	<0.001
Glycochenodeoxycholic acid	2.19	<0.001
Deoxycholic acid	−2.32	<0.001

## Data Availability

The data that support the findings of this study are available from the corresponding author, [D.M.], upon reasonable request.

## Supplementary Materials

The following supporting information can be downloaded at: https://www.mdpi.com/article/10.3390/cancers17071150/s1, File S1: Additional participants demographics; File S2: PCA raw data; File S3: PCA adjusted data; File S4: VIF calculations for all NMR metabolites; File S5: VIF calculations following adjusting for multicollinearity; File S6: Significant metabolites and calculations; File S7: Mass spectrometry heatmap.
