# Supplementary figures and images for: Metabolite Changes Associated with Resectable Pancreatic Ductal Adenocarcinoma
Source: Cancers (Basel). 2025 Mar 29;17(7):1150. doi: 10.3390/cancers17071150 (PMC11988049; doi:10.3390/cancers17071150)

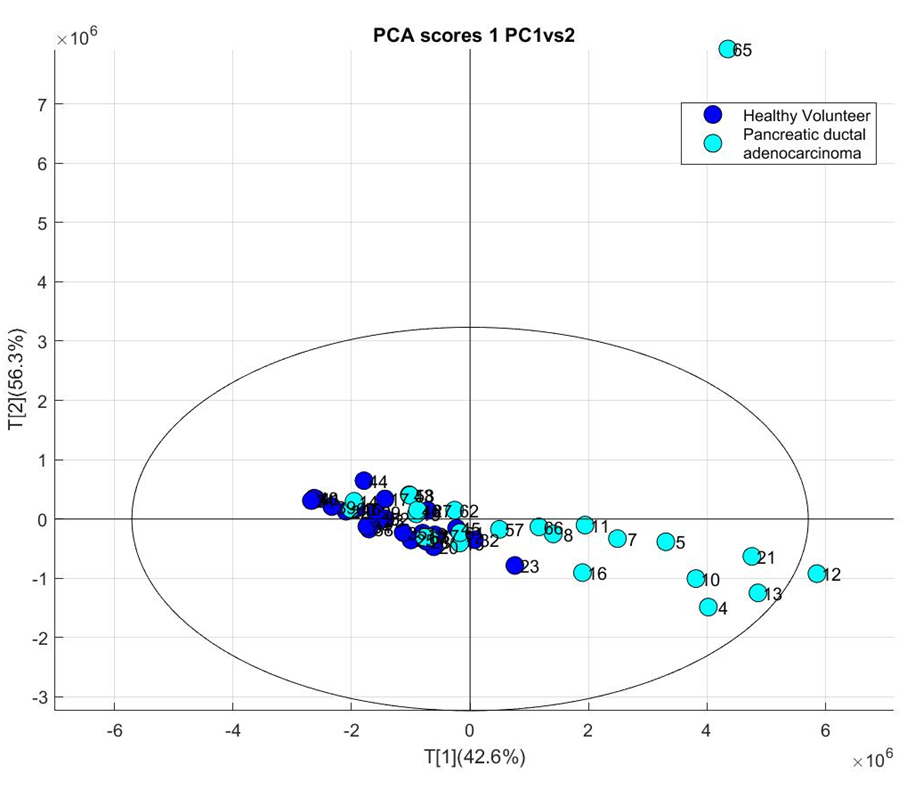

Supplement: Supplementary file 1 [file cancers-17-01150-s001.zip › S2 PCA raw data.png]

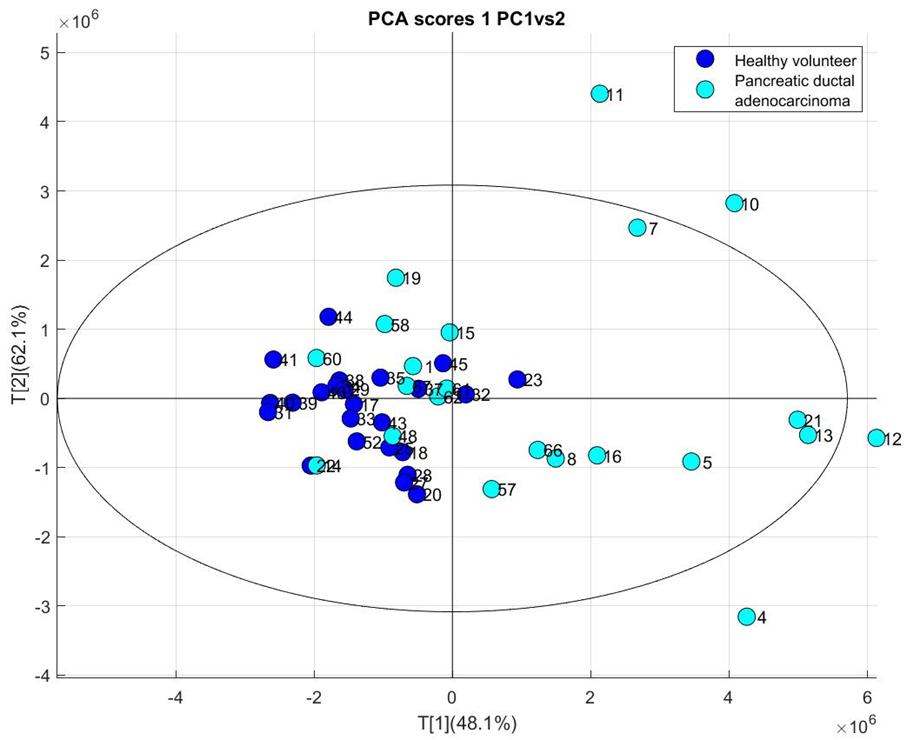

Supplement: Supplementary file 1 [file cancers-17-01150-s001.zip › S3 PCA adjusted data.png]

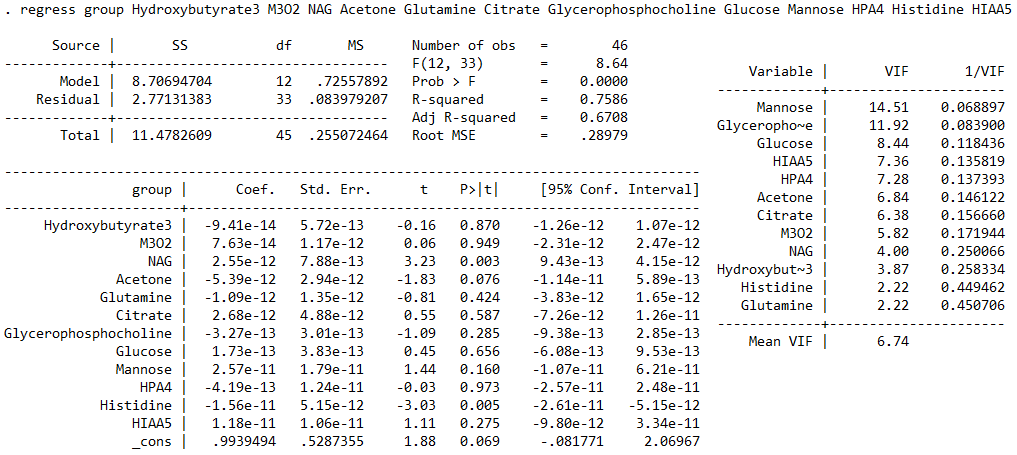

Supplement: Supplementary file 1 [file cancers-17-01150-s001.zip › S4 VIF calculations for all NMR metabolites.png]

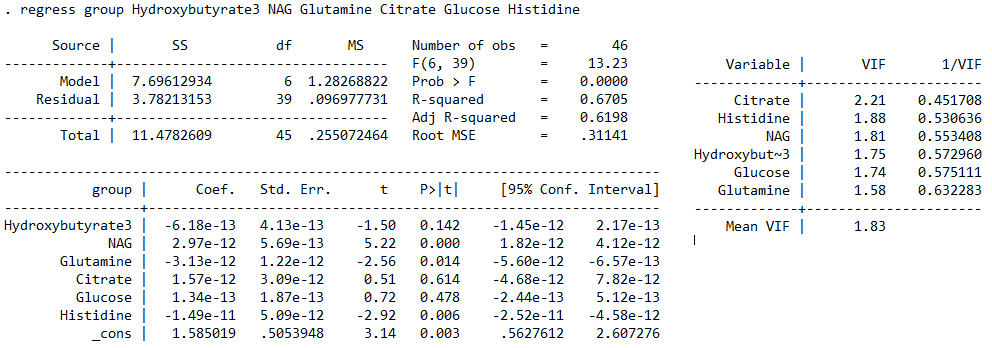

Supplement: Supplementary file 1 [file cancers-17-01150-s001.zip › S5 VIF calculations following adjusting for multicollinearity.png]

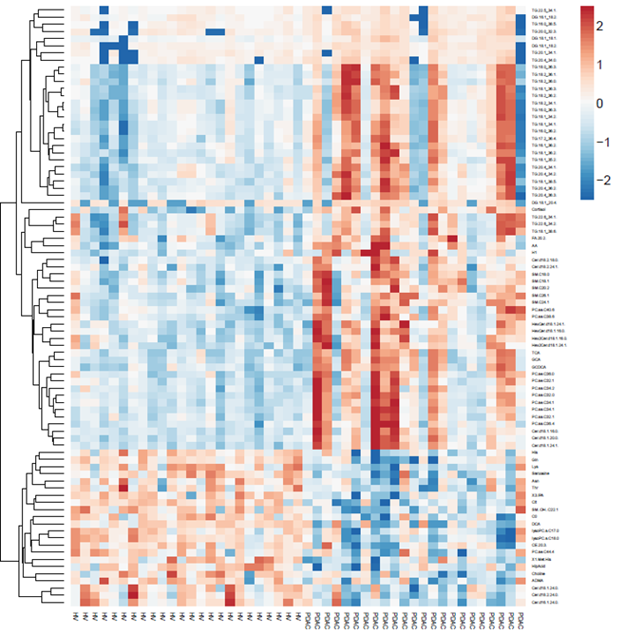

Supplement: Supplementary file 1 [file cancers-17-01150-s001.zip › S7 Mass spectrometry heatmap.png]
